# Exopolysaccharide-Derived Carbon Dots for Microbial Viability Assessment

**DOI:** 10.3389/fmicb.2018.02697

**Published:** 2018-11-09

**Authors:** Fengming Lin, Chengcheng Li, Zhan Chen

**Affiliations:** ^1^State Key Laboratory of Bioelectronics, School of Biological Science and Medical Engineering, Southeast University, Nanjing, China; ^2^Department of Chemistry, University of Michigan, Ann Arbor, MI, United States

**Keywords:** carbon-based nanomaterial, polysaccharides, microbial viability assessment, biocompatibility, photostability

## Abstract

Fluorescent dye staining combined with fluorescence microscopy or flow cytometry is becoming a routine way to monitor microorganism viability that is necessary for food safety, antibiotic development, and human health. However, the conventional live/dead assay dyes suffer from high cost, inconvenient staining steps, and high cytotoxicity, which is urgently needed to overcome. Herein, cheap carbon dots, CDs-EPS605, were reported to successfully assess microbial viability in a convenient way with neglectable cytotoxicity. The fluorescent N-doped CDs-EPS605 could be facilely prepared from bacterial amino exopolysaccharide (EPS) by one-step hydrothermal carbonization, which is cost-effective and sustainable. The negatively charged CDs-EPS605 consisted of C, H, O, N, P, and S, and featured various functional groups, including -COOH, -OH, -CONH-, and -NH_2_. CDs-EPS605 were observed to sensitively and selectively stain dead microorganisms instead of live ones to enable discrimination of live/dead microorganisms. The labeling method with CDs-EPS605 did not require protection from light, or washing, which is convenient. Additionally, CDs-EPS605 displayed better photostability and much less cytotoxicity compared to the commercial counterpart. Altogether, CDs-EPS605 represent a simple, yet powerful staining agent for microbial viability assessment, and at the same time enrich the current applications of microbial EPS.

## Introduction

Microbial viability assessment is commonly performed during pathogen detection, antibiotic development and microbial monitoring in both industrial and biomedical fields (Breeuwer and Abee, 2000). It is also a routine assay in microorganism-related study in the lab. Conventionally, microbial viability assessment was carried out by plate counting that is time-consuming and labor-intensive with low sensitivity and large deviation (Zhao et al., 2014). Other methods like atomic force microscopy (AFM) (Fantner et al., 2010), fourier transform infrared spectroscopy (FT-IR) (Notingher et al., 2003), surface-enhanced raman scattering (Zhou et al., 2015) and nucleic acid sequence-based amplification (Keer and Birch, 2003) have been under development to gain higher accuracy, but they are time-consuming and costly, requiring pretreatment of organisms. Compared to these methods, fluorescent dye staining paired with fluorescence microscopy or flow cytometry is far more used for the differentiation of live/dead microorganism due to that it is simple, fast, sensitive, and visible (Breeuwer and Abee, 2000; Zhao et al., 2014). However, the commercial dyes of this fluorescence-based method, such as propidium monoazide (PMA) and propidium iodide (PI), that selectively label dead cells, suffer from high cost, toxicity, unstability, photobleaching, and cumbersome protocol (Breeuwer and Abee, 2000; Zhao et al., 2014). Therefore, developing desirable dyes for microbial viability evaluation is of great value.

Carbon dots (CDs) are commonly carbon nanoparticles with sizes less than 10 nm that may also contain oxygen, hydrogen, and other elements. CDs are well known for their high water-dispersity, superior photostability, good biocompatibility, and low cost, making them attractive photoluminescent nanomaterials. They have found a wide range of biological applications including bioimaging, biosensing, thermometer, photothermal therapy, photodynamic therapy, and drug delivery (Reckmeier et al., 2016). For bioimaging, they have been successfully utilized to discriminate gram positive/negative bacteria (Yang et al., 2016), and live/dead microorganisms (Hua et al., 2017). The amphiphilic C-dots have been synthesized and employed as an excellent tool for labeling biomimetic and cellular membranes (Nandi et al., 2014, 2015), and imaging biofilm matrix (Ritenberg et al., 2016). CDs synthesized from *Lactobacillus plantarum by* one-step hydrothermal carbonization were used successfully for imaging the biofilm-encased microorganisms (Lin et al., 2017).

Exopolysaccharides (EPSs) secreted out of microorganisms are sugar biopolymers consisting of homo- or hetero-saccharides that are linked together by glycosidic bonds to generate long linear or branched chains. The naturally occurring EPSs are abundant, readily available, biodegradable, and potentially non-toxic (Moscovici, 2015; Bhatia, 2016). EPSs have found a vast range of applications in nanotechnology, like green synthesis of metal nanoparticles (Kanmani and Lim, 2013; Sathiyanarayanan et al., 2017), hydrogel (Na et al., 2000; Maiti et al., 2011; Koop et al., 2015), coating materials (Jang et al., 2013; You et al., 2014; Bondarenko et al., 2016), stabilizers (Dey et al., 2016), nanoparticles (Wang et al., 2013; Guo et al., 2014; Zhang et al., 2016), capping agents (Wu et al., 2012; Yan et al., 2015). Even more, EPS can self-assembled into different nanostructures like nanofibers (Xu et al., 2013; Chen et al., 2016) and nanoparticles (Li et al., 2017), which could be employed as promising bio-active carriers for DNA (Liu et al., 2014, 2015), protein (Takahashi et al., 2014), and drug (Takedatsu et al., 2012). They can be further modified into nanocomplexes for drug delivery applications. Though many bio-macromolecules including cellulose, chitosan, proteins, and gelatin have used as molecular precursors of CDs (Reckmeier et al., 2016), so far there are few study on bacterial EPS-based CDs.

In this study, fluorescent N-doped CDs were successfully prepared by a facile hydrothermal method using bacterial amino EPS as the precursors. The microstructure, chemical compositions, optical properties of these CDs were investigated. The as-synthesized CDs have been demonstrated to be capable of discriminating live/dead microorganisms, which was evaluated and compared to the commercial counterpart in terms of universality, photostability, and biocompatibility.

## Materials and Methods

*Lactobacillus plantarum* LLC-605 was obtained from the traditional Chinese fermented food FuYuan Pickles (Yunnan, China) with excellent exopolysaccharide-producing ability in our lab (Li et al., 2017). *E. coli, S. aureus, M. luteus, B. subtilis, and P. pastoris* were ordered from China Center of Industrial Culture Collection (CICC, Beijing, China). Extracellular polysaccharide EPS-605 was purified from the fermentation broth of *L. plantarum* LCC-605, as described in our previous study (Li et al., 2017). Dialysis membranes (Spectra/Por6 Dialysis membranes, Regenerated Cellulose) with a molecular weight cutoff of 1 kDa were purchased from Sangon Biotech. Co., Ltd. (Shanghai, China). Lysogeny broth (LB), potato dextrose agar (PDA), and potato dextrose broth (PDB) were ordered from Beijing Land Bridge Technology (Beijing, China).

### CDs Preparation

CDs were fabricated from the purified EPS-605 by one-step hydrothermal carbonization. EPS-605 was dissolved in 10 mL Milli-Q water to reach the final concentration of 2 mg/mL, transferred into a 50 mL Teflon-lined stainless-steel autoclave, and then incubated at 200°C for 24 h. The resulting dark brown solution was cooled down at room temperature and centrifuged at 12000 rpm for 10 min. The supernatant was filtered through a 0.22 μm filter membrane to further remove large particles/aggregates. At last, the CD solution was dialyzed in a 1 kDa cutoff membrane against Milli-Q water for 48 h, and stored at 4°C for further use.

### Characterizations of CDs

The produced CDs from EPS-605 were characterized in terms of the size, morphology and element components. A drop of CD solution was deposited on a 400-mesh carbon-coated copper grid and examined using a transmission electron microscope (JEM-2100, JEOL Ltd., Japan). Ultraviolet-visible (UV-vis) and fluorescence spectra of CDs in pure water or phosphate-buffered saline (PBS: 137 mM NaCl, 2.7 mM KCl, 10.1 mM Na_2_HPO_4_, 1.7 mM KH_2_PO_4_, pH 7.4) were measured by a UV–vis spectrophotometer (UV-2600, Shimadzu, Japan) and a spectrofluorophotometer (RF-5301PC, Shimadzu, Japan), respectively. Fourier transform infrared (FTIR) spectroscopy of CDs was obtained on an FTIR spectrometer (Nicolet iS50, Thermo Scientific, United States). X-ray photoelectron spectroscopic (XPS) experiment was carried out with a Japan Kratos Axis Ultra HAS spectrometer. Zeta potential values of CDs were measured using a Zetasizer instrument (Malvern Instruments, Nano ZS, United Kingdom). The fluorescence QY of the CDs was calculated according to the method previously reported (Yang et al., 2015; Pramanik et al., 2016a,b). Briefly, a comparative assay was utilized using the following equation (Yang et al., 2015):

$$\emptyset_{\text{c}}=\emptyset_{\text{s}}\times \frac{I_{\text{c}}}{I_{\text{s}}}\times \frac{A_{\text{s}}}{A_{\text{c}}}\times \frac{\eta_{\text{c}}}{\eta_{\text{s}}}$$

Where ∅, *I, A*, and η presents the fluorescence quantum yield, the integrated area in the emission spectrum, UV-vis absorbance at the fluorescent excitation wavelength, and the refractive index of the solvent, respectively. The subscripts “c” and “s” denote the CDs solution and the standard solution of quinine sulfate in 0.1 M H_2_SO_4_, respectively. A_c_ and A_s_ obtained in a 1 cm cuvette should be less than 0.1 to avoid the reabsorption effect. The QY of a quinine sulfate solution excited at 306 nm is reported to be 54% (Yang et al., 2015).

### Differentiation of Live/Dead Microorganisms

Bacteria were grown in LB at 37°C for 14–18 h, while fungi were cultured in PDB at 32°C for 24–48 h. Dead cells were prepared by boiling at 100°C for 30 min. Both live and dead cells were incubated with 0.8 mg/mL CDs-EPS605 for 90 min without light protection, and then observed immediately on a fluorescent confocal microscope (TCS SP8, Leica, Germany) without washing. The excitation wavelengths were 405, 488, and 552 nm with the corresponding emissions detected in the range of 405–490, 500–565, and 570–630 nm, respectively.

### Comparison Between CDs-EPS605 and PI on the Live/Dead Cell Assessment

The imaging performance of CDs-EPS605 and the commercial dye PI was compared. A mixture of live and dead *S. aureus* was incubated with the mixture of CDs-605 (800 ug/mL) and PI for 90 min in dark, and then imaged under a confocal microscope. PI has an excitation/emission maxima of 488/615 nm. The imaging conditions were finely tuned to realize that only the desired dye PI showed red fluorescence under the excitation wavelength of 488 nm, so that the effect of the spectral overlap between CDs-EPS605 and PI in the co-labeling experiments was avoided. Namely, under the tested conditions in this study, the red fluorescence intensity of CDs-EPS605 was much lower than that of PI, so we reduced the excitation power to achieve that CDs-EPS605 did not display red fluorescence that was evidenced with dead cell samples stained only with CDs-EPS605, while PI still had red fluorescence that was demonstrated with dead cell samples labeled with CDs-EPS605 alone.

### Photostability Assay

For the UV irradiation experiment, the corresponding PL intensities of the CD solutions were recorded after irradiation under a UV lamp of 365 nm (20 mW) for different times as indicated in the text. The CD solutions with different pH values (3, 5, 7, 9, and 11) were made with the pH adjusted via concentrated HCl or NaOH solution, whose PL intensities were assayed at 500 nm (λ_ex_ = 460 nm) on the spectrofluorophotometer. The PL intensities of the CD solutions at various temperatures (4, 25, 40, 55, 70, 85, and 100°C) and different buffer concentrations (0, 10, 100, 200, 300, 400, 500, 600, 700, 800, and 900 mM) were measured. The same experiments were performed for the small molecular dye PI as a comparison.

### Effect of CDs-EPS605 on Microorganism Growth

Both *E. coli* and *S. aureus* were grown in LB overnight with 180 rpm at 37°C, while *P. pastoris* was grown in PDA with 180 rpm at 32°C. The cell culture was diluted 1:100 in PDA or LB with different concentrations of CDs-EPS605 (0, 0.1, 0.2, 0.4, 0.8, 1, and 3 mg/mL), of which 200 μL/well was transferred into the 96-well plates (Costar, Corning, United States). The inoculated 96-well plates were incubated for 24 h at 37°C with 180 rpm. Then, the OD of the cell cultures was recorded at 600 nm.

Colony forming unit (CFU) counting method was performed to further study the cytotoxicity of CDs-EPS605 to *E. coli*. Briefly, *E. coli* in log phase were grown in LB in the presence of 3 mg/mL CDs-EPS605 at 37°C with 180 rpm for 24 h. Each culture was diluted with LB with a dilution factor of 1 × 10^5^. The diluted microbial culture were plated on LB agar plates in triplicate and incubated at 37°C for 24 h. Then, the cell colonies of *E. coli* were measured. Three biological replicates were carried out. The cytotoxicity was determined by comparing the number of CFUs in treated groups to that in the control group.

## Results and Discussion

### Synthesis and Characterization of Carbon Dots

**FIGURE 1:**
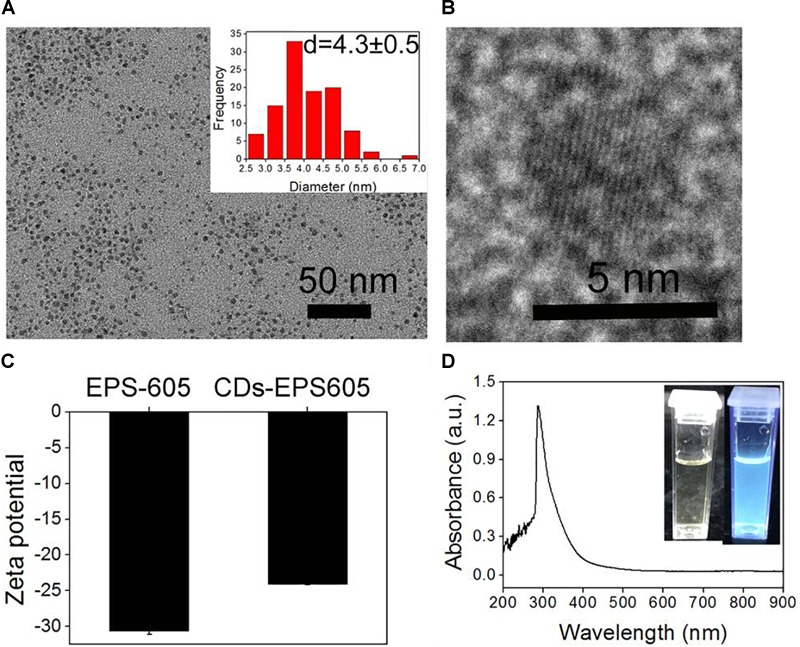
Profiling of CDs-EPS605. **(A)** TEM image of CDs-EPS605. Inset: the corresponding size histogram. **(B)** The high resolution TEM of CDs-EPS605. **(C)** The zeta potential of CDs-EPS605. **(D)** The UV-Vis absorption spectra of CDs-EPS605 in H_2_O. Inset: photographs of CDs-EPS605 in H_2_O without and with the UV irradiation at 365 nm.

**FIGURE 2:**
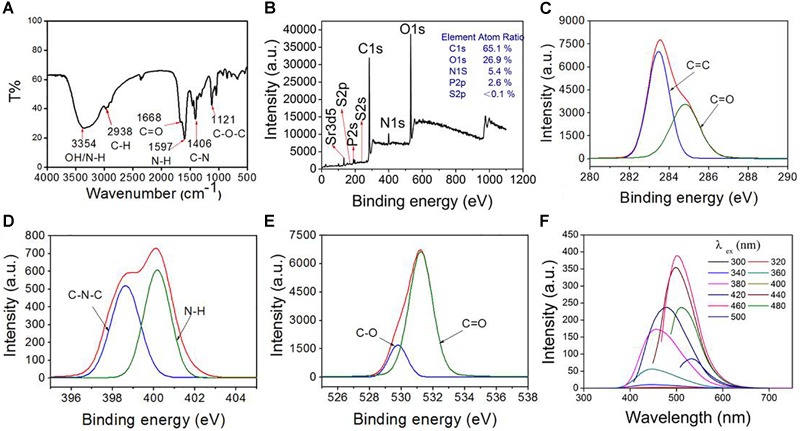
The compositions of CDs-EPS605 were determined by FTIR **(A)** and XPS **(B)**. The high-resolution XPS peaks of C1s **(C)**, N1s **(D)**, and O1s **(E)**. **(F)** Fluorescence spectra of CDs-EPS605 in water.

We considered microbial exopolysaccharide EPS-605 as an excellent raw material for CDs synthesis, given that it contains elements C, H, O, S, P, and N (Li et al., 2017), providing both the major carbon framework and other elements for doping to produce CDs without the utilization of passive agents (Reckmeier et al., 2016). To prove this, purified EPS-605 from strain *L. plantarum* LLC-605 was exploited to prepare CDs by one-step hydrothermal reaction. The as-produced CDs were referred to as CDs-EPS605. The TEM image showed that CDs-EPS605 were well monodispersed and quasi-spherical, with an average diameter of 4.3 nm (Figure 1A). In the high-resolution image of CDs-EPS605 (Figure 1B), a crystalline structure was observed. CDs-EPS605 were negatively charged with a zeta potential of -24 mV (Figure 1C), owing to that the raw material EPS-605 has a highly negative charge of -32.6 mV (Li et al., 2017). CDs-EPS605 exhibited strong blue fluorescence when excited at 365 nm with an ultraviolet lamp (Figure 1D), demonstrating CDs-EPS605 were well dispersed in aqueous solution. The UV-VIS absorption spectrum of CDs-EPS605 exhibited a huge absorption peak at 320 nm (Figure 1D), which might be due to the n-π^∗^ transition of C = O group on the surface of CDs-EPS605 (Figures 2A,E; Song et al., 2017). Except this, no characteristic absorption peak was found as reported in other carbon nanomaterials (Zhao et al., 2011; Jiang et al., 2017; Lin et al., 2017).

### Compositions of CDs-EPS605

The compositions of CDs-EPS605 were detected by FTIR and XPS. The broad absorption band around 3354 cm^-1^ was assigned to the stretching vibrations of O-H [ν(O-H)] and/or N-H [ν(N-H)] (Figure 2A). The peak at 1668 cm^-1^ belonged to the C = O stretching vibration, ν(C = O), due to the -COOH group or the amide bond (-CONH-). The peak at 1597 cm^-1^ was attributed to the N-H bending vibration δ(N-H), due to the NH_2_ group or the amide bond (-CONH-). The peak at 1406 cm^-1^ might originated from the O-H/C-H bending vibration [δ(O-H)/δ(C-H)], and the one at 1121 cm^-1^ was owning to the C-O stretching vibrations [ν(C-O)].

CDs-EPS605 contains the elements of C (65.1%), O (26.9%), N (5.4%), P (2.6%), and S (<0.1%) as determined by XPS (Figure 2B). In the high-resolution C1s spectrum (Figure 2C), the two fitted peaks at 284.6 and 285.9 eV were attributed to carbon-containing groups C-C/C = C (sp2 carbons) and C-N, respectively (Bao et al., 2015). The N1s peak at 399.7 and 401.3 eV (Figure 2D) were separately ascribed to amide nitrogen C-N-C and the amino nitrogen N-H (Yang et al., 2014; Zhang and Chen, 2014). For the O1s spectrum, the two fitted peaks at 530.9 and 532.3 eV (Figure 2E) were attributed to the oxygen element in the forms of C = O and C-O, respectively (Zhang and Chen, 2014; Ding et al., 2016). Overall, the FTIR and XPS results demonstrated that the as-fabricated CDs-EPS605 contains the elements C, H, O, S, P, and N, and the functional groups -OH, -COOH, -CONH-, and -NH_2_. These findings show that the functional groups of the starting material EPS-605 are conferred onto CDs-EPS605 (Li et al., 2017).

### Optical Properties of CDs-EPS605

When the excitation wavelength was changed from 360 to 420 nm or from 440 to 500 nm, no red-shift was observed for the emission wavelength. Clearly, the fluorescence emission spectrum of CDs-EPS605 only partly relies on the excitation wavelength. The emission intensity of CDs-EPS605 rose steadily from 300 nm to 460 nm with a peak at 460 nm, and then fell from 480 nm to 520 nm (Figure 2F). The maximum fluorescence emission intensity was found at 500 nm with an excitation wavelength of 460 nm. The QY of CDs-EPS was 3.86%. Obviously, CDs-EPS605 were N-doped and multi-colored luminescent.

The starting material for CDs-EPS605, exopolysaccharide EPS-605, can be readily obtained from *L. plantarum* LCC-605 that is abundant, renewable, and biocompatible. Meanwhile, the synthesis method is simple and facile. Compared to other EPSs derived from animals like chitin or from plants like cellulose and gelatin that have been employed as precursors for production of CDs (Chowdhury et al., 2012; Shchipunov et al., 2015; Shen et al., 2016), the production of microbial EPS from fermentation is timesaving and controllable with much higher production titer (Moscovici, 2015). The defined and reproducible EPS production by easily controlled fermentation parameters would ensure a high quality of CDs-EPS production. By contrast, using biomass for CD fabrication such as galic (Zhao et al., 2015), fruit juice(Sahu et al., 2012) and bacteria (Hua et al., 2017), whose components is very complicated and difficult to be profiled, suffer from uncontrolled quality from batch to batch (Reckmeier et al., 2016). Furthermore, EPS605 contains 7% element N as measured by XPS (Li et al., 2017) and thus no dopants or surface passivation agents is required for the synthesis of N-doped CDs-EPS605. When using polysaccharides without N element, dopants such as urea (Shen et al., 2016) and ethylenediamine (Chen et al., 2017) were added to introduce into CD structures N element that has a favorable effect on the CD optical properties. Other N-containing polysaccharides like chitin and chitosan have also been used as raw materials for CD production (Chowdhury et al., 2012; Shchipunov et al., 2015). Nevertheless, these two polysaccharide suffer from poor water solubility, and strong acid is used like nitric acid (Shchipunov et al., 2015) and acetic acid (Chowdhury et al., 2012) in the hydrothermal reaction. Notably, EPS605 represent an ideal precursor for the production of green N-doped CDs for high sustainability, readily availability, controlled quality, N-containing, and good water solubility.

### CDs-EPS605 Can Discriminate Live/Dead *S. aureus*

**FIGURE 3:**
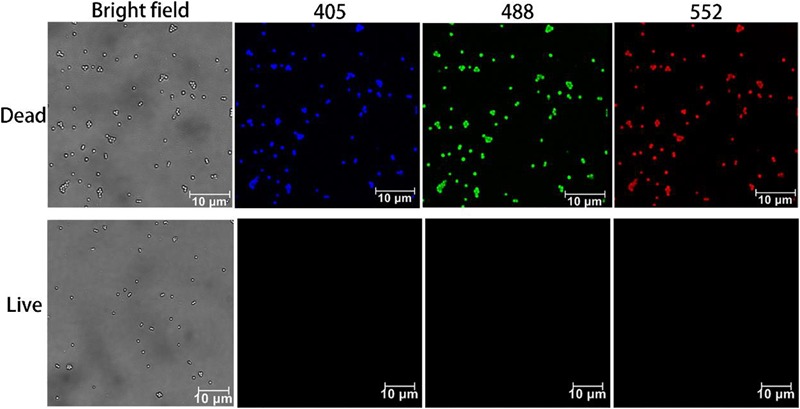
Confocal images of live and dead *S. aureus* labeled with CDs-EPS605. Fluorescence images were obtained under the excitation of 405, 488, and 552 nm, respectively. Bright field images were also presented for reference.

Both live and dead *S. aureus* were incubated with CDs-EPS605 without light protection and then examined under confocal laser scanning microscopy (CLSM) without washing (Figure 3). According to the fluorescence spectra of CDs-EPS605 (Figure 2F), the sample was viewed in three channels including blue, green and red when excited at 405, 488, and 552 nm, respectively. No fluorescence was observed for the live and healthy *S. aureus* cultured at 37°C overnight, indicating they were not labeled by CDs-EPS605. In contrast, strong fluorescence was detected in all three tested channels for dead *S. aureus* killed by boiling for 10 min, demonstrating that the dead cells were stained by CDs-EPS605. Therefore, CDs-EPS605 can be used to discriminate between live/dead *S. aureus* cells. Meanwhile, the three-color imaging of dead *S. aureus* of CDs-EPS605 enables flexibility in terms of the laser options for excitation.

**FIGURE 4:**
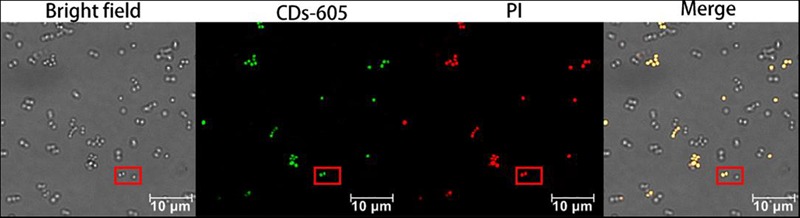
Confocal fluorescence images of live and dead *S. aureus* co-labeled with CDs-EPS605 and PI. The excitation wavelength was 488 and 552 nm for CDs-EPS605 and PI, respectively. In the red squares, two dead *S. aureus* cells were co-labeled by both CDs-EPS605 and PI, while one live *S. aureus* cell was not stained.

To further confirm the live/dead microorganism discrimination ability of CDs-EPS605, a mixture of live and dead *S. aureus* was co-stained by CDs-EPS605 and the commercial live/dead assay dye PI that is well-known for selectively labeling dead cells but not live ones to distinguish live/dead cells. The fluorescence of CDs-EPS605 co-localized well with that of PI, and live *S. aureus* were not labeled by either PI or CDs-EPS605 (Figure 4), confirming that CDs-EPS605 could only stain dead *S. aureus*.

### CDs-EPS605 Possess Excellent Photostability

**FIGURE 5:**
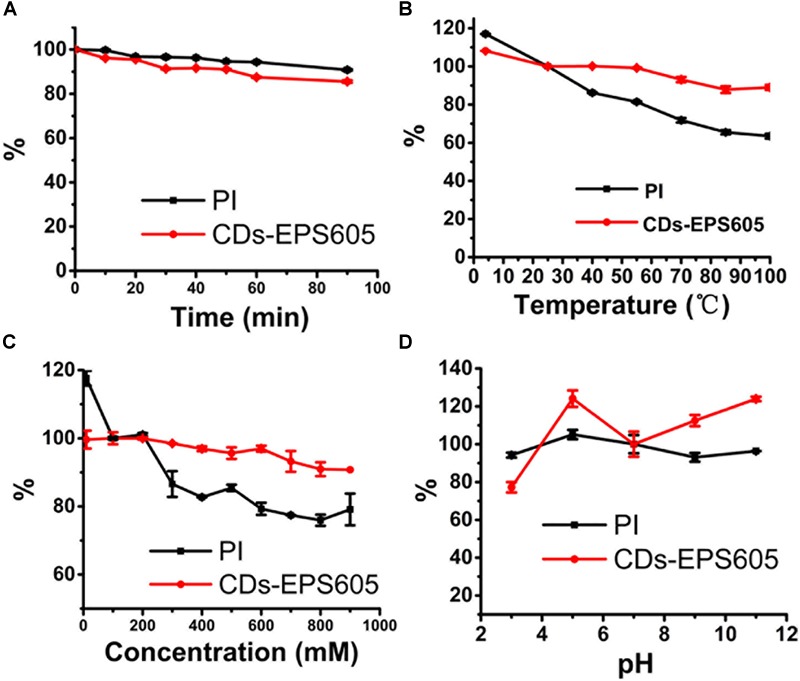
PL properties of CDs-EPS605 as a function of UV irradiation time **(A)**, temperature **(B)**, ionic strength (different concentrations of PBS solution, pH = 7.4) **(C)** and pH **(D)**. The PL intensity was measured at 514 nm (λex = 488 nm). The PL intensity of CDs-605 in PBS (pH = 7.4) without laser irradiation was measured at 25°C and was arbitrarily assigned as 100%.

Good photostability of dyes under various experimental conditions is important and valuable for practical applications. Hence, the effect of UV irradiation time, temperature, buffer concentration and pH on the photoluminescence (PL) property of both CDs-EPS605, and the commercial dye PI in solutions, was evaluated (Figure 5). The PL of both CDs-EPS605 and PI had a slight decrease under the continuous UV irradiation for 90 min (Figure 5A). The PL of CDs-EPS605 remained almost unchanged as the buffer concentration or the temperature was increased, which is different from that the PL of PI was reduced noticeably (Figures 5B,C). At pH 3–11, a larger extend of fickleness of PL was observed for CDs-EPS605 than PI, indicating CDs-EPS was more sensitive to pH than PI (Figure 5D). In all tested conditions, at least 80% PL of CDs-EPS605 was maintained, showing the excellent photostability of CDs-EPS605 under various environment conditions (Figure 5). Obviously, the photostability of CDs-EPS605 is better than PI, making the live/dead microorganism assessment more flexible and convenient.

### The Live/Dead Cell Distinction by CDs-EPS605 Is Microorganism-Universal

**FIGURE 6:**
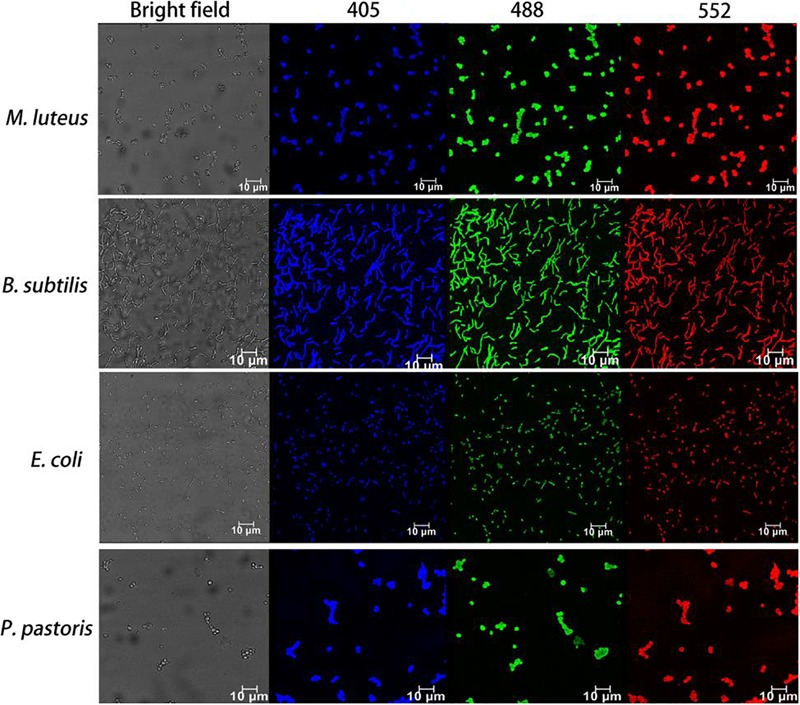
Fluorescence images of dead microorganisms covering two other Gram-positive bacteria (*M. luteus* and *B. subtilis*), one Gram-negative bacterium (*E. coli*) and yeast (*P. pastoris)* labeled with CDs-EPS605. The corresponding bright field was also presented. Fluorescence images were observed with the excitation length of 405, 488, and 552 nm, respectively.

The universality of the live/dead cell differentiation with CDs-EPS605 is explored by incubating CDs-EPS605 with different bacteria, both dead and live, including *M. luteus* (Gram positive), *S. subtilis* (Gram positive), *E. coli* (Gram negative), and fungus *P. pastoris* (Figure 6 and Supplementary Figure S1). For all tested microorganisms, only dead microorganisms show strong florescence in the blue, green and red channels excited with 405, 488, and 552 nm, respectively (Figure 6), whereas no fluorescence was observed for the corresponding live ones (Supplementary Figure S1). These findings demonstrate CDs-EPS605 could selectively stain dead microorganisms regardless of their species and can be applied to discriminate between live and dead microorganisms.

### CDs-EPS605 Are Non-toxic Toward Microorganisms

To assess the toxicity of CDs-EPS605 on microorganisms, the effect of CDs-EPS605 on microorganism growth was figured out by determining the optical density of cell culture at 600 nm (OD600). *S. aureus, E. coli*, and *P. pastoris*, each as the model for Gram-positive bacteria, Gram-negative bacteria, and fungi, respectively, were chosen for the evaluation. The OD600 of cell culture from these three microorganisms remained unaltered when grown for 24 h in the presence of CDs-EPS605 of 0, 0.1, 0.2, 0.4, 0.8, 1, and 3 mg/mL. This implied that CDs-EPS605 had no toxicity toward microorganisms. This result was further proved by the plate counting result using *E. coli* as an example (Supplementary Figure S2). It is reported that PI caused a significant toxicity toward both *S. aureus* and yeast at its working concentration of 30 μM, leading to significantly reduced cell viability of these two microorganisms treated with 30 μM PI for 24 h as compared to the untreated control (Hua et al., 2017). Hence, CDs-EPS605 are more suitable for long-time, accurate labeling of dead microbes than PI.

**FIGURE 7:**
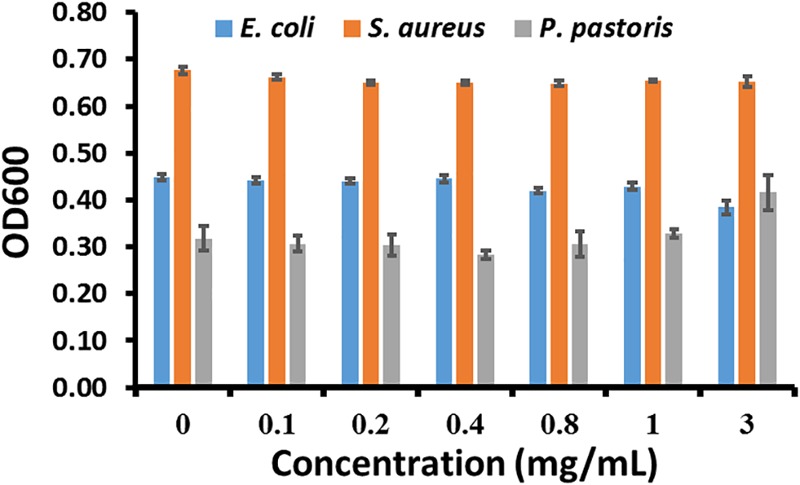
The effect of CDs-EPS605 on growth of microorganisms. The OD600 of *E. coli, S. aureus, and P. pastoris* for 24 h was measured, respectively in culture media containing 0–3 mg/mL CDs-EPS605 at 37°C with 180 rpm.

Currently, the expensive commercial dyes for live/dead assay require picky storage condition (-20°C), sample avoidance from light when staining, and sample washing after the staining, which was not required for CDs-EPS605. The starting material EPS-605 can be generated abundantly by fermentation of *L. plantarum* using MSA as the carbon source with a high yield of 940 mg/L (Li et al., 2017). MSA is sold at ∼45 $/250 g in China. Meanwhile, the hydythermal reaction is done at 200°C for 24 h, which is also a cheap process. Based on these, CD-EPS605 is considered cost-effect. Furthermore, CDs-EPS605 featured prominent photostability (Figure 5) and no cytotoxicity (Figure 7). Obviously, the discrimination of live/dead microorganisms by CDs-EPS605 is easier and safer than those commercial counterparts, while it is also cheap.

Through zeta potential measurement, FTIR, and XPS, it was found that the main surface group of CDs-EPS605 is the negatively charged carboxyl group, leading to a strong electrostatic repulsion interaction between CDs-EPS605 and the negatively charged microorganisms. Additionally, there are hydrophilic groups such as -COOH and -OH on CDs-EPS605, preventing the hydrophobic interaction between CDs-EPS605 and microorganisms. Such highly negative and hydrophilic surface of CDs-EPS605 impedes them from penetrating into the live microorganisms. In contrast, the ultrasmall size of CDs-EPS605 can enter the dead microorganisms with compromised cell walls and plasma membranes, due to that the integrity of these microorganisms’ walls and membranes might be damaged or lost (Strauber and Muller, 2010), achieving the selective staining of these dead ones.

## Conclusion

In this study, we reported amino EPS from lactate acid bacteria (LAB) was first time explored as a single precursor to produce fluorescence N-doped CDs viz. CDs-EPS605 through simple one-step hydrothermal reaction without any dopants and passivation agents. The participating amino EPS derived from fermentation of *L. plantarum* is readily produced and renewable, making the synthesis route green and eco-friendly. Characterization of CDs-EPS605 showed that CDs-EPS605 had an average diameter of 3.1 nm and display good dispersibility. CDs-EPS605 consisting of the elements C, H, O, N, P, and S with the functional groups -OH, -CONH-, -NH_2_, and -COOH, were negatively charged and carry multicolor under excitation at different wavelengths. More important, CDs-EPS605 were found to be capable of selectively labeling dead microbial cells but not live ones, which can be applied in the microbial viability assay. Compared to the current live/dead assay dyes such as PI, CDs-EPS605 possessed fascinating merits including multicolor fluorescence emission property, negligible cytotoxicity, excellent photostability, low cost, and convenient staining process. Furthermore, the synthetic strategy of CDs-EPS605 is facile, eco-friendly and economic, enabling the large-scale production of live/dead microbial differentiation kits based on CDs-EPS605, which would broaden the researches of microorganism detection. Our study advances the applications of bacterial EPSs and offers a new, renewable, and eco-friendly dye CDs-EPS605 as an excellent alternative to current commercial live/dead microbial assay dyes.

## Author Contributions

FL conceived and designed the study. FL carried out the majority of the experiments. CL conducted the preparation of EPS-605 and CDs-EPS605. CL, FL, and ZC analyzed the data and drafted the manuscript. All authors read and approved the final manuscript.

## Conflict of Interest Statement

The authors declare that the research was conducted in the absence of any commercial or financial relationships that could be construed as a potential conflict of interest. The reviewer NKR and handling Editor declared their shared affiliation.
