# Supplementary material for: Exopolysaccharide-Derived Carbon Dots for Microbial Viability Assessment
Source: Front Microbiol. 2018 Nov 9;9:2697. doi: 10.3389/fmicb.2018.02697 (PMC6237930; doi:10.3389/fmicb.2018.02697)
Supplement: Supplementary file 1 [file Data_Sheet_1.docx]

**Supporting information for publication**

**
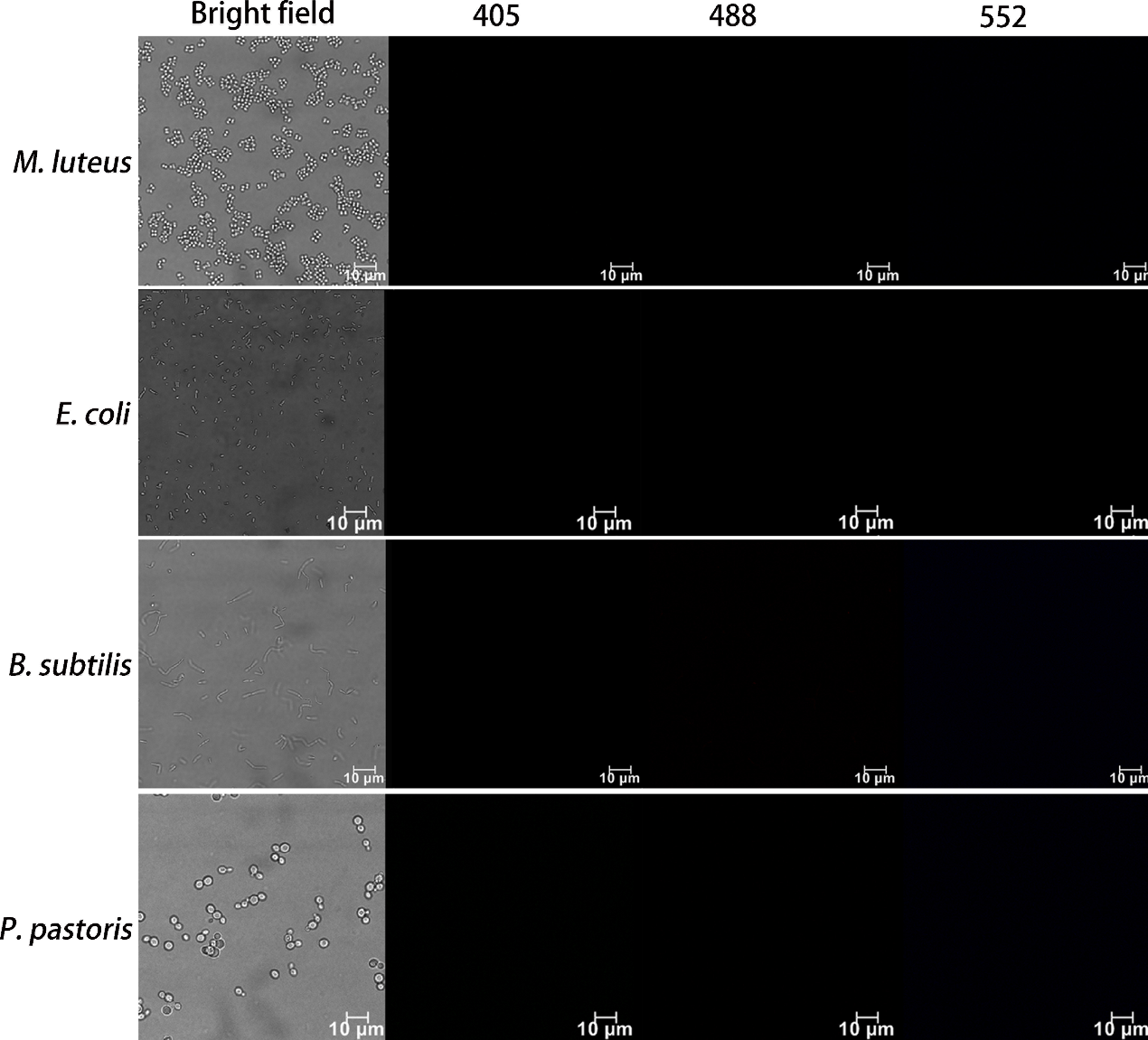
**

Figure S1 Fluorescence images of live microorganisms including two other Gram-positive bacteria (*M. luteus* and *B. subtilis*), one Gram-negative bacterium (*E. coli*) and yeast (*P. pastoris)* stained with CDs-EPS605. Fluorescence images were captured under the excitation of 405, 488 and 552 nm, respectively. The corresponding bright field was also presented.


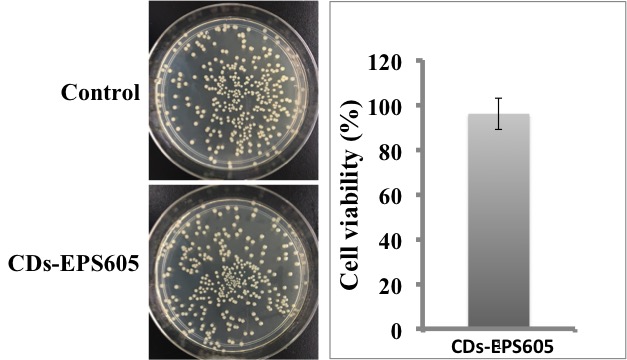


Figure S2 Photographs of the agar plates and the corresponding statistical histogram of colonies of *E. coli* cultured in LB (control) and CDs-EPS605 (3mg/mL) for 24 h.
